# Chronic stress in mice remodels lymph vasculature to promote tumour cell dissemination

**DOI:** 10.1038/ncomms10634

**Published:** 2016-03-01

**Authors:** Caroline P. Le, Cameron J. Nowell, Corina Kim-Fuchs, Edoardo Botteri, Jonathan G. Hiller, Hilmy Ismail, Matthew A. Pimentel, Ming G. Chai, Tara Karnezis, Nicole Rotmensz, Giuseppe Renne, Sara Gandini, Colin W. Pouton, Davide Ferrari, Andreas Möller, Steven A. Stacker, Erica K. Sloan

**Affiliations:** 1 Drug Discovery Biology Theme, Monash Institute of Pharmaceutical Sciences, Monash University, Parkville, Victoria 3052, Australia; 2 Department of Visceral Surgery and Medicine, University Hospital Bern, 3010 Bern, Switzerland; 3 Division of Epidemiology and Biostatistics, European Institute of Oncology, Milan 20141, Italy; 4 Department of Cancer Anaesthesia and Pain Medicine, Peter MacCallum Cancer Centre, East Melbourne, Victoria 3002, Australia; 5 Peter MacCallum Department of Oncology, The University of Melbourne, Parkville, Victoria 3010, Australia; 6 Tumor Angiogenesis Program, Peter MacCallum Cancer Centre, East Melbourne, Victoria 3002, Australia; 7 Division of Pathology and Laboratory Medicine, European Institute of Oncology, Milan 20141, Italy; 8 Drug Delivery, Disposition and Dynamics Theme, Monash Institute of Pharmaceutical Sciences, Monash University, Parkville, Victoria 3052, Australia; 9 Department of Mathematics and Statistics, The University of Melbourne, Parkville, Victoria 3010, Australia; 10 Cancer Division, QIMR Berghofer Medical Research Institute, Herston, Queensland 4006, Australia; 11 Cousins Center for PNI, University of California Los Angeles, Los Angeles, CA 90095, USA; 12 UCLA Semel Institute, University of California Los Angeles, Los Angeles, CA 90095, USA; 13 Jonsson Comprehensive Cancer Center, University of California Los Angeles, Los Angeles, CA 90095, USA; 14 UCLA AIDS Institute, University of California Los Angeles, Los Angeles, CA 90095, USA

## Abstract

Chronic stress induces signalling from the sympathetic nervous system (SNS) and drives cancer progression, although the pathways of tumour cell dissemination are unclear. Here we show that chronic stress restructures lymphatic networks within and around tumours to provide pathways for tumour cell escape. We show that VEGFC derived from tumour cells is required for stress to induce lymphatic remodelling and that this depends on COX2 inflammatory signalling from macrophages. Pharmacological inhibition of SNS signalling blocks the effect of chronic stress on lymphatic remodelling *in vivo* and reduces lymphatic metastasis in preclinical cancer models and in patients with breast cancer. These findings reveal unanticipated communication between stress-induced neural signalling and inflammation, which regulates tumour lymphatic architecture and lymphogenous tumour cell dissemination. These findings suggest that limiting the effects of SNS signalling to prevent tumour cell dissemination through lymphatic routes may provide a strategy to improve cancer outcomes.

## Abstract (web summary)

Adverse life events have been associated with reduced survival in cancer patients. Here, the authors explore the mechanism responsible and show that chronic stress in mice activates a signalling cascade in macrophages and tumour cells, which results in restructuring of the tumour lymphatic system, promoting metastasis.

## Graphical abstract

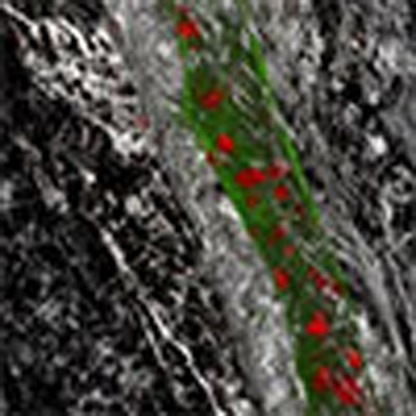

In everyday life, we encounter stressful experiences that pose a threat to physiological homeostasis. These threats trigger stress responses, including activation of the sympathetic nervous system (SNS), which leads to elevated local and systemic levels of catecholaminergic neurotransmitters that signal to cells^1^. Stress-induced SNS signalling is important to enhance alertness and physiological functions for rapid reaction to threat^2^. However, chronic periods of stress can be detrimental to health by increasing inflammation and promoting the progression of diseases including cancer^3^,^4^,^5^,^6^. Clinical studies have linked experience of stressful events to poor cancer survival^7^,^8^. This is supported by preclinical studies that show chronic stress promotes cancer progression^3^,^4^,^6^. These studies found that stress recruits inflammatory cells to tumours and increases the formation of blood vessels^3^,^6^, which may provide routes for tumour cell dissemination. In addition to dissemination through blood vessels, cancer cells also escape from tumours through lymphatic vasculature^9^,^10^,^11^.

The lymphatic system plays an important role in immune function and therefore can influence the trajectory of disease progression. Under normal physiological conditions, the lymphatic system maintains homeostasis by directing cells and solutes from the interstitial fluid of peripheral tissues through lymphatic vessels and into lymph nodes, where they undergo immune examination^12^,^13^. In addition, the lymphatic system aids in the resolution of inflammation by transporting immune cells away from sites of infection^14^. In cancer, the lymphatic system contributes to disease progression by providing a pathway for tumour cell escape while also being a rich source of chemokines that can promote the invasive properties of tumour cells^15^. Furthermore, tumour-draining lymph nodes and associated lymphatic endothelium have been shown to develop an immunosuppressive environment, which promotes immune tolerance to the cancer and facilitates tumour growth and spread^16^,^17^,^18^. The importance of the lymphatic system in cancer progression is supported by vast clinical data that show tumour-associated lymphatic vessel density (LVD), tumour cell invasion into lymphatic vasculature and the presence of tumour cells in lymph nodes are each associated with increased clinical tumour stage and reduced disease-free survival^19^,^20^,^21^.

The lymphatic system is innervated by fibres of the SNS^22^, and acute SNS activity has been shown to increase lymphatic vessel contraction^23^,^24^ and lymphocyte output into lymphatic circulation^25^. However, little is known about whether stress-induced SNS signalling affects tumour lymphatic vasculature and the consequences this may have on cancer progression.

In this study, we show that chronic stress increases intratumoural LVD while also inducing dilation and increasing flow in lymphatic vessels that drain metastatic tumour cells into lymphatic circulation. Inhibition of COX2 activity blocked the effect of stress on lymphatic vascular remodelling, and showed a key role for macrophage-mediated inflammation in the effects of stress. In addition, we show a critical role for tumour cell-derived VEGFC in the effects of stress on lymphatic vasculature. In both clinical and preclinical studies we demonstrate that disrupting SNS regulation of lymphatics, by blocking β-adrenoceptor signalling, protects against lymphatic dissemination and cancer progression. These findings identify stress signalling as a regulator of lymphatic remodelling and provide evidence for the feasibility of clinically targeting SNS regulation of lymphatics to prevent tumour cell dissemination through lymphatic routes.

## Results

### Chronic stress remodels tumour lymphatic vasculature

**Figure 1:**
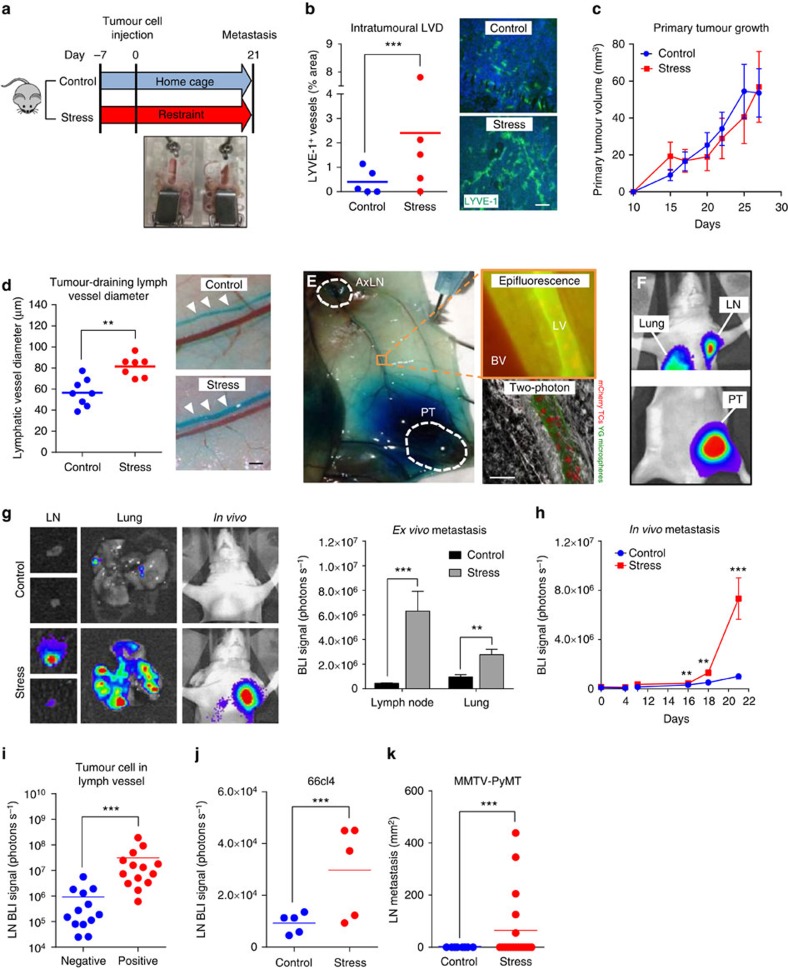
Chronic stress remodels tumour-associated lymphatic architecture to promote lymph node metastasis. (**a**) Schematic representation of the chronic stress paradigm. (**b**) Quantification and representative images of tumour LVD (LYVE-1^+^, green; nuclear, blue) immunostaining of MDA-MB-231 orthotopic tumours. Scale bar, 200 μm (*n*=5). (**c**) Quantification of MDA-MB-231 primary tumour size in control or stressed BALB/c *nu/nu* mice over time (*n*=5 at each time point). (**d**) Quantification and representative images of tumour-draining lymphatic vessel diameter (LV, blue) in mice with MDA-MB-231 tumours. Scale bar, 1 mm (*n*⩾7). (**e**) Left: skin flap preparation after injection of Patent Blue V dye into the primary tumour (PT) showing the dye taken up into the tumour-draining LV and into the tumour-draining axillary lymph node (AxLN). The LV is adjacent to a blood vessel (BV). Right top panel: epifluorescence image of mCherry-tagged MDA-MB-231 tumour cells (TCs, red) that had spontaneously disseminated from orthotopic PT and were present in the tumour-draining LV that contained microspheres (green) and was adjacent to an autofluorescent BV. Right lower panel: corresponding maximum projection of multiphoton image. Scale bar, 100 μm (Supplementary Movie 1). (**f**) Representative *in vivo* bioluminescence image of orthotopic MDA-MB-231 breast cancer model showing PT, and spontaneous metastasis to draining lymph node (LN) and lung 21 days after tumour cell injection. (**g**) Representative images of LN and lung metastasis and quantification of metastasis by *ex vivo* bioluminescence (BLI) imaging in control versus stressed mice with MDA-MB-231 tumours (*n*=5). (**h**) Metastasis *in vivo* over time (*n*=5 at each time point). (**i**) LN metastasis in mice that were negative or positive for tumour cells in collecting lymphatic vessels (*n*⩾13). (**j**) *Ex vivo* quantification of bioluminescence from LN at day 28 of 66cl4 tumour progression from control or stressed mice (*n*=5). (**k**) Area of lymph node metastasis when primary tumour diameter reached 12 mm in control or stressed MMTV-PyMT mice (*n*⩾8). Experiments were completed 2–4 times. All data represent mean±s.e. **P<0.01 and ***P<0.001 by Student's *t*-test or Mann–Whitney *U*-test (*post hoc* Bonferroni correction).

Stress-related psychosocial factors have been linked to increased cancer-related mortality^8^. This is supported by accumulating preclinical data that show chronic stress acts through SNS signalling to promote progression of multiple tumour types^3^,^4^,^6^,^26^. However, the role of the lymphatic system in stress-induced tumour cell dissemination is unknown. To examine the effect of stress on tumour-associated lymphatics, we used an orthotopic model of breast cancer in which primary tumours were developed from MDA-MB-231 human breast cancer cells. Mice were subjected to a well-established chronic stress paradigm that activates SNS signalling whereby inescapable confinement induces escape-avoidance behaviour (Fig. 1a). This paradigm has been shown to elevate stress hormones without activating motor-sensory pain pathways^3^. To examine the effect of stress on intratumoural lymphatic vasculature, we used immunohistochemical analysis to quantify the expression of the lymphatic endothelial cell marker LYVE-1 (ref. 27) within primary tumour sections as a measure of tumour LVD. Multiple fields of view were captured around the periphery of the tumour margin (Supplementary Fig. 1A). Chronic stress significantly increased tumour LYVE-1 staining compared with control tumour-bearing mice (Fig. 1b), demonstrating that stress-induced SNS signalling can increase the lymphatic network within primary tumours. Consistent with our previous findings, chronic stress did not affect primary tumour growth (Fig. 1c and Supplementary Fig. 1B), confirming that the effect of stress on LVD was independent of primary tumour size. Stress similarly increased tumour LVD in immunocompetent BALB/c mice with 66cl4 luminal B mammary tumours (Supplementary Fig. 1C) demonstrating that stress induces lymphatic remodelling in a model with an intact immune system.

Our previous studies found that dilation of lymphatic vessels that drain primary tumours increase metastasis to lymph node^28^. We showed that lymph vessel dilation was dependent on inflammatory signalling, but the physiological conditions in which collector dilation increases metastasis remain unclear^29^. Therefore, we sought to evaluate if, in addition to increasing intratumoural LVD, chronic stress could also change the diameter of lymphatic vessels that drain primary tumours. We used the clinical lymph-tracing agent Patent Blue V dye^30^ to track lymph drainage from the primary tumour and to quantify the diameter of tumour-draining lymphatic vessels^28^. Comparisons between the lymphatic vessels of control versus stressed mice revealed that chronic stress caused significant dilation of the lymphatic vessel that drains the primary tumour (Fig. 1d). This effect was independent of changes in the diameter of the adjacent blood vessel (Supplementary Fig. 1D) and was maintained even 2 weeks after the cessation of stress, showing that stress induces stable changes in tumour-associated lymphatic architecture. These data demonstrate the ability of chronic stress signalling to not only increase the density of lymphatic vessels within the primary tumour but also to induce stable dilation of the lymphatic vessels that drain primary tumours, suggesting an increased capacity for tumour cell dissemination.

As the functionality of intratumoural lymphatics has been debated^31^,^32^, we first examined if tumour-associated lymphatic vessels are viable routes for tumour cell dissemination. Immunohistochemical analysis of primary mammary tumours identified cell emboli within the lumen of intratumoural lymphatic vessels that stained positive for LYVE-1 (Supplementary Fig. 1E). Furthermore, we show that the tumour-associated lymphatic vasculature in this model is functional as it drained Patent Blue V dye from the tumour (Fig. 1d,e). To investigate if tumour cells spontaneously disseminate from orthotopic mammary tumours into lymphatic vasculature, we developed methodology to directly visualize tumour cells in these tumour-draining lymphatic vessels in live animals. MDA-MB-231 tumour cells were tagged with the mCherry fluorescent marker and implanted into the fourth mammary fat pad (Fig. 1e, left panel). Once tumours reached 150 mm^3^, fluorescently tagged nanospheres were injected into the inguinal lymph node, and intravital multiphoton fluorescence microscopy was used to visualize the nanospheres flowing through the draining lymphatic vessel *in situ* (Fig. 1e, right panels). We detected mCherry^+^ tumour cells that had spontaneously intravasated from the primary tumour along the length of the draining lymphatic vessel towards the tumour-draining lymph node (Fig. 1e, Supplementary Fig. 1F and Supplementary Movie 1).

Having demonstrated that lymphatic vasculature is indeed a route for spontaneous tumour cell dissemination in this model, we then sought to determine whether these stress-induced alterations in lymphatic architecture were associated with increased tumour cell dissemination to lymphatic organs. We used MDA-MB-231 cells tagged with firefly luciferase for longitudinal *in vivo* optical bioluminescence imaging to monitor cancer progression (Fig. 1f). Consistent with tumour LVD and lymphatic vessel dilation having a key role in lymphogenous tumour cell dissemination^28^, stress-induced changes in lymphatic architecture were associated with increased metastasis to lymph nodes (Fig. 1g). Chronic stress also accelerated initial signs of metastasis, which were observed as early as 11 days post tumour cell injection (Fig. 1h and Supplementary Fig. 1G). The presence of tumour cells in the tumour-draining lymphatic vessel was associated with increased lymph node metastasis (Fig. 1i), and there was a shift to higher frequency of tumour-positive lymph nodes with stress (Supplementary Fig. 1H and Supplementary Table 2). Consistent with clinical observations that lymph node involvement is prognostic for distant metastasis, tumour cell dissemination to lymph nodes was associated with increased distant metastasis to lung (Fig. 1g). Chronic stress similarly increased tumour cell dissemination to lymph nodes in immunocompetent BALB/c mice with 66cl4 mouse mammary tumours^33^ and in mouse mammary tumour virus-polyoma middle T oncoprotein (MMTV-PyMT) transgenic mice that spontaneously develop mammary tumours and metastasis (Fig. 1j,k)^34^. These findings identify chronic stress as a regulator of tumour-associated lymphatics and demonstrate that stress-induced changes in lymphatic architecture are functional and provide pathways for tumour cell dissemination to increase lymph node metastasis. While these findings do not exclude that stress also increases metastasis by promoting haematogenous routes of dissemination^3^,^6^, they raise the possibility that targeting stress signalling to modulate lymphatic vasculature may limit lymphatic avenues of tumour cell dissemination.

### SNS signalling can be leveraged to regulate lymph flow

**Figure 2:**
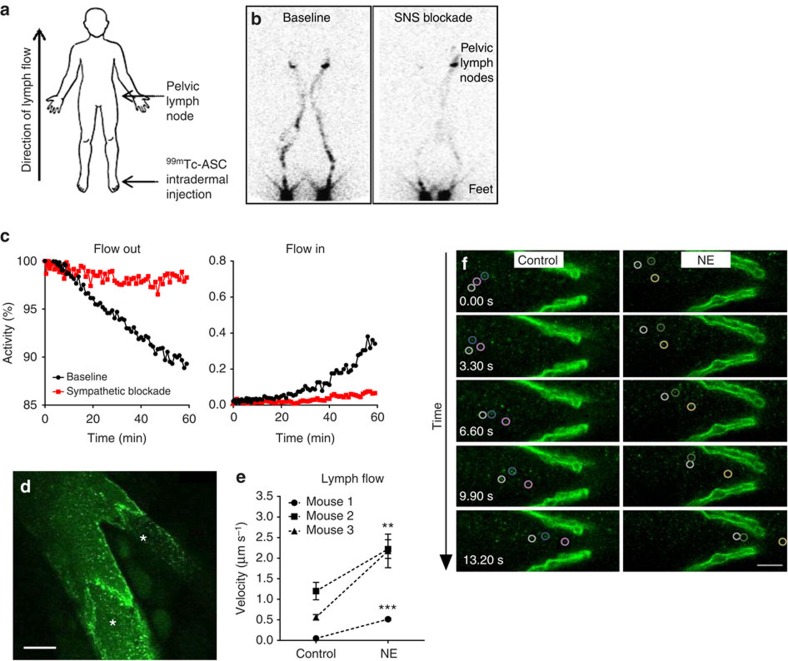
SNS signalling may be targeted to limit lymph flow. (**a**) Schematic representation of lymph flow measurement in patients. ^99m^Tc-ASC was injected into feet and tracked to pelvic lymph nodes using nuclear lymphoscintigraphy. (**b**,**c**) Quantification and lower limb lymphoscintigram of ^99m^Tc-ASC washout from feet and wash-in to pelvis at baseline or after SNS blockade, taken 60 min after injection (*n*=1). (**d**) Multiphoton image of nanospheres (green) used to track flow through collecting lymphatic vessel and branching capillary. Asterisks indicate valves. Scale bar, 50 μm. (**e**) Quantification and (**f**) representative images of lymph velocity through collecting lymphatic vessels of mice before (control) or after norepinephrine (NE) treatment (*n*=3). Scale bar, 20 μm (Supplementary Movie 2). All data represent mean±s.e. **P<0.01 and ***P<0.001 by Student's *t*-test.

Innervation of the lymphatic system by fibres of the SNS provides evidence for a direct link between the stress-activated SNS and lymphatic function. This raises the possibility that SNS signalling could increase lymph flow and facilitate tumour cell dissemination. Using multiphoton microscopy, we first confirmed that lymph nodes are innervated with catecholaminergic TH^+^ fibres of the SNS (Supplementary Fig. 2A). To investigate if the SNS could be acutely targeted to modulate lymphatic function, we examined the effect of neuraxial anaesthesia on lymph flow in a clinical setting. Some anaesthetic agents, such as neuraxial anaesthesia, inhibit nerve signalling, which results in sympathectomy in the area of associated sensory blockade^35^. Therefore, to investigate whether acute inhibition of sympathetic signalling could reduce lymph flow, we conducted a proof-of-concept study using nuclear lymphoscintigraphy to track the effect of neuraxial anaesthesia on lymph flow in the lower limbs of a consenting patient receiving brachytherapy for cervical carcinoma. Unlike most patients receiving spinal anaesthesia, patients receiving brachytherapy have no surgery-induced lower limb wound oedema that would otherwise confound interpretation of the study's findings. Regional sympathetic blockade was induced by the administration of intrathecal ropivicaine at L3/4. Nuclear lymphoscintigraphy was used to track lymph drainage of a radiolabelled tracer (Technetium-99m antimony sulphide colloid, ^99m^Tc-ASC) from the injection site in the feet through lymph vasculature to lymph nodes in the pelvis (Fig. 2a). To minimize confounding of anatomical variation, lymph flow was assessed before and after the implementation of SNS blockade in the same individual. To minimize the potential contribution of blood pressure, heart rate or muscle contraction to changes in lymph drainage, the patient remained stationary in a supine position for the duration of the study, and lymphoscintigraphy only commenced after blood pressure was stabilized. Under baseline conditions, tracer activity at the site of injection in the feet decreased over time and accumulated in pelvic lymph nodes, indicating clearance of the tracer by lymphatic flow (Fig. 2b,c). In contrast, sympathetic blockade reduced outflow of the tracer from feet by 84% and inflow to pelvic lymph nodes by 80% after 60 min, demonstrating that SNS signalling is necessary for lymph flow and that clinical strategies may be used to modulate SNS signalling and block lymph function.

Having shown that acutely blocking SNS signalling blocks lymph flow, we then investigated if conversely activating SNS signalling was sufficient to increase lymph flow. Activation of the SNS leads to the release of the endogenous neurotransmitter norepinephrine from local SNS fibres^1^. To mimic the local release of norepinephrine from tissue nerve endings, norepinephrine was administered intravenously to directly activate SNS signalling pathways, and lymph flow was measured using intravital multiphoton microscopy to track the rate of transit of fluorescent nanospheres through lymphatic vessels of anaesthetized mice. Nanospheres were injected into the inguinal lymph node and the baseline rate of flow of nanospheres through the draining efferent lymphatic vessel was quantified after 3 min had elapsed to allow flow to stabilize. Recording from lymphatic vessels was confirmed by the presence of occasional valves (Fig. 2d). Treatment with a single dose of norepinephrine significantly elevated the rate of lymph flow through collecting lymphatic vessels in mice (Fig. 2e,f and Supplementary Movie 2) demonstrating the ability of acute SNS signalling to promote lymphatic flow. The baseline rate of lymph flow in the absence of norepinephrine treatment was observed to vary considerably between animals (Supplementary Fig. 2B), consistent with previous data^36^. Therefore, as with the clinical lymph flow studies, measurements were taken in each mouse before and after norepinephrine administration. Together, these findings indicate that SNS signalling is a potent regulator of lymph flow and that existing clinical strategies may be utilized to selectively alter lymph function.

### β-adrenergic signalling remodels tumour lymphatic vessels

**Figure 3:**
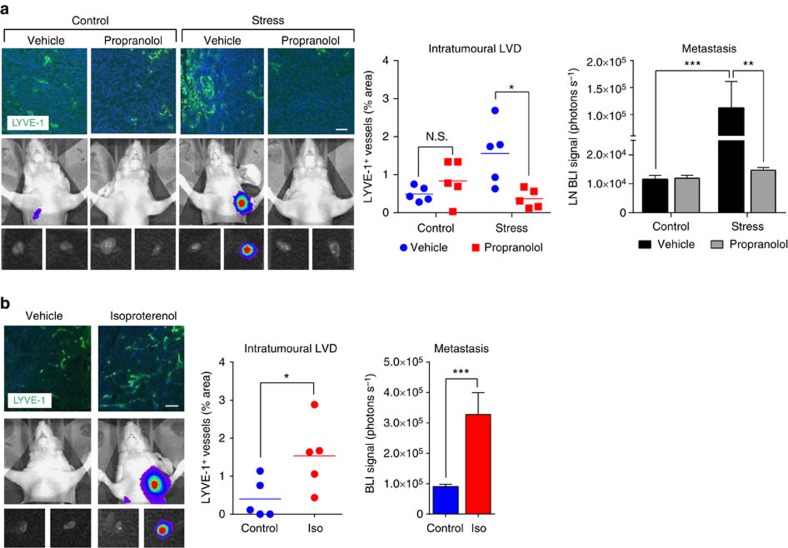
β-adrenergic signalling is necessary for stress-induced remodelling of tumour LVD and lymphatic metastasis. (**a**) Representative images and quantification of tumour LVD (LYVE-1^+^, green; nuclear, blue) immunostaining and bioluminescence imaging of vehicle- or propranolol-treated mice with MDA-MB-231 primary tumours 22 days post tumour cell injection (*n*=5). Scale bar, 200 μm. N.S.=not significant. (**b**) Representative images and quantification of tumour LVD (LYVE-1^+^, green; nuclear, blue) immunostaining and bioluminescence imaging of vehicle- or isoproterenol (Iso)-treated mice with MDA-MB-231 primary tumours (*n*=5). *P<0.05, **P<0.01 and ***P<0.001 by Mann–Whitney *U*-test or two-way analysis of variance (*post hoc* Tukey's adjustment).

Norepinephrine is released from parenchymal nerve fibres in response to stress, and binds β-adrenoceptors to modulate cell function^1^. β-adrenoceptors are present on tumour cells and stromal cell populations in the tumour microenvironment^37^,^38^, including inflammatory cells^6^. Accumulating evidence from preclinical studies suggests that chronic stress promotes cancer progression through β-adrenoceptor activation^3^,^4^,^6^,^26^,^39^. To investigate if β-adrenergic signalling is required for the effects of stress on intratumoural LVD, we treated mice with propranolol, a non-selective β-adrenoceptor antagonist (beta-blocker; BB) that is used clinically for the treatment of hypertension. Treatment with propranolol during MDA-MB-231 tumour development blocked chronic stress from increasing tumour LYVE-1^+^ LVD and reduced metastasis to lymph nodes, suggesting that reduced LVD had functional effects on tumour cell dissemination (Fig. 3a and Supplementary Table 1). Conversely, systemic activation of β-adrenoceptors with the β-adrenoceptor agonist isoproterenol was sufficient to increase intratumoural LVD and increase lymph node metastasis (Fig. 3b and Supplementary Fig. 3A) without affecting primary tumour growth (Supplementary Fig. 3B). These findings demonstrate that β-adrenoceptor signalling is sufficient to promote intratumoural LVD and is necessary for the adverse effects of stress on lymphatic vascular remodelling.

### VEGFC mediates stress-induced lymphatic remodelling

**Figure 4:**
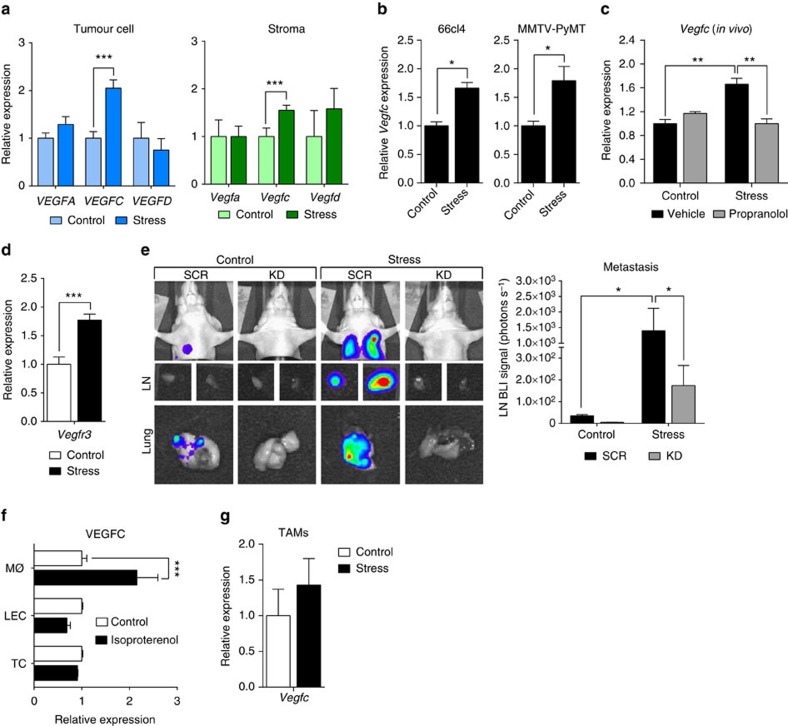
Tumour cell-derived VEGFC is necessary for stress-induced lymphatic metastasis. (**a**) qRT–PCR analysis of tumour cell (human)- or stroma (mouse)-specific *VEGF* gene expression in MDA-MB-231 primary tumours of control versus stressed mice (*n*=5). Expression normalized to *RPL30*. (**b**) qRT–PCR analysis of *Vegfc* gene expression in 66cl4 and MMTV-PyMT primary tumours. Expression normalized to *Rpl30* (*n*=5). (**c**) qRT–PCR analysis of *Vegfc* gene expression in 66cl4 primary tumours of control or stressed mice treated with propranolol (*n*=5). Expression normalized to *Rpl30*. (**d**) qRT–PCR analysis of mouse-specific *Vegfr3* (*Flt4*) gene expression in MDA-MB-231 primary tumours (*n*=5). Expression normalized to *Rpl30*. (**e**) Representative images and quantification of bioluminescence from mice with MDA-MB-231 tumours expressing *VEGFC* short hairpin RNA (knockdown, KD) or scrambled (SCR) control (*n*=5). (**f**) qRT–PCR analysis of *VEGFC* gene expression in MDA-MB-231 tumour cells (TCs), human LECs or primary macrophages (MØ) treated with isoproterenol or vehicle (*n*=3). Expression normalized to *RPL30.* (**g**) qRT–PCR analysis of *Vegfc* gene expression in CD11b^+^F4/80^+^ TAMs isolated from 66cl4 tumours in control versus stressed mice (*n*=3). *P<0.05, **P<0.01 and ***P<0.001 by Student's *t*-test or two-way analysis of variance (*post hoc* Tukey's adjustment).

To define the molecular mechanisms that drive stress-induced changes in lymphatic vasculature, we used quantitative reverse transcription–PCR (qRT–PCR) to profile the effect of stress on the expression of vascular endothelial growth factors (VEGFs). VEGFA, VEGFC and VEGFD are central to lymphangiogenesis, and their expression in tumours is associated with poor cancer outcome^21^,^40^,^41^,^42^. Using species-specific probes (Supplementary Fig. 4A), we found that chronic stress elevates the expression of both tumour cell-derived and stromal cell-derived *VEGFC* in MDA-MB-231 primary tumours (Fig. 4a). SNS regulation of *VEGFC* expression was also confirmed in 66cl4 mammary tumours from immunocompetent mice or in mammary tumours from MMTV-PyMT transgenic mice (Fig. 4b). VEGFC has been shown to promote tumour lymphangiogenesis and metastasis to lymph nodes in preclinical cancer models^43^,^44^, and is associated with increased lymph node metastasis and decreased progression-free survival in patients^40^. Treatment with isoproterenol also elevated tumour cell and stromal cell *VEGFC* expression (Supplementary Fig. 4B), while BB treatment blocked the effect of stress on *VEGFC* expression (Fig. 4c), demonstrating the importance of β-adrenoceptor activation in stress-induced expression of this lymphangiogenic factor. VEGFC binds to its receptor VEGFR3 (FLT4) to drive tumour lymphangiogenesis^43^,^45^,^46^,^47^. Chronic stress or treatment with isoproterenol also increased stromal cell expression of *Vegfr3* (Fig. 4d and Supplementary Fig. 4C), indicating that β-adrenoceptor signalling increases the capacity for VEGFC–VEGFR3 signalling.

To investigate the contribution of tumour cell-derived VEGFC in SNS-induced lymphatic remodelling, we used short hairpin RNA to stably silence *VEGFC* in MDA-MB-231 tumour cells (Supplementary Fig. 4D). Knockdown of VEGFC blocked stress-induced LYVE-1^+^ LVD in primary tumours (Supplementary Fig. 4E), demonstrating a key role for tumour cell *VEGFC* in stress-induced lymphatic remodelling. *VEGFC* knockdown also blocked the ability of stress to promote lymphogenous metastasis and distant metastasis (Fig. 4e), consistent with a critical role for *VEGFC*-dependent lymphangiogenesis in stress-enhanced tumour cell dissemination. These data demonstrate that tumour cell-derived VEGFC is necessary for stress-induced lymphatic remodelling and metastasis.

To begin to investigate possible stromal sources of VEGFC, we treated lymphatic endothelial cells (LECs) and primary macrophages—both of which express β-adrenoceptors (Supplementary Fig. 4F)^6^,^48^—with isoproterenol. β-agonism increased *Vegfc* expression in macrophages but not in LECs (Fig. 4f). However, stress did not elevate *Vegfc* expression in CD11b^+^F4/80^+^ macrophages isolated from primary mammary tumours (Fig. 4g), suggesting that there may be additional stromal sources of stress-regulated VEGFC.

### Stress remodels tumour lymphatics via inflammation

**Figure 5:**
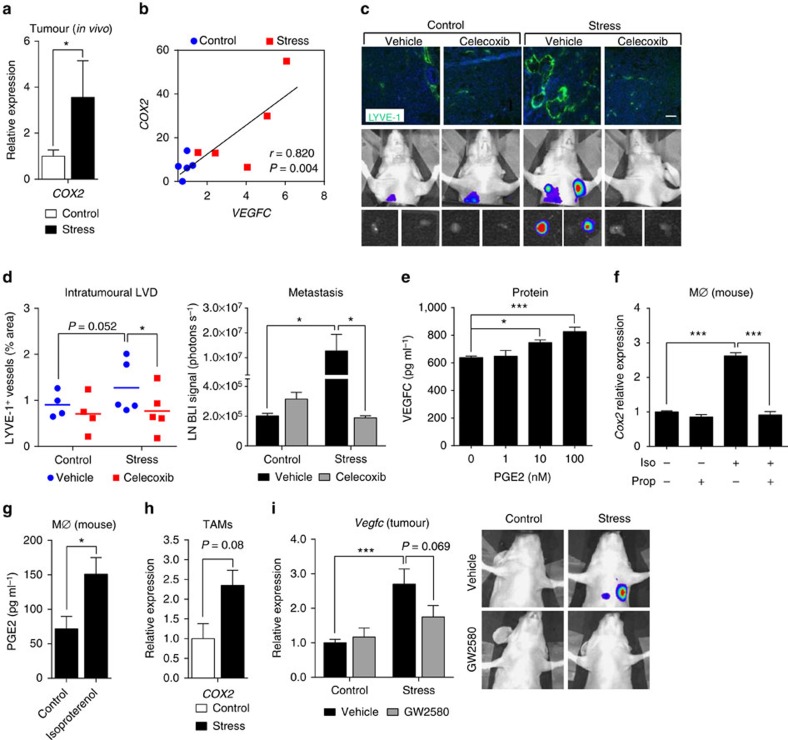
Inflammatory signalling is required for stress-induced lymphatic remodelling. (**a**) qRT–PCR analysis of *COX2* gene expression in MDA-MB-231 primary tumours of control versus stressed mice. Expression normalized to *RPL30* (*n*=5). (**b**) Relationship between *COX2* and *VEGFC* expression in MDA-MB-231 tumours of control or stressed mice (*n*=5). Pearson coefficient 0.820 (*P*=0.004). (**c**) Representative images and (**d**) quantification of LVD (LYVE-1^+^, green; nuclear, blue) immunostaining of MDA-MB-231 primary tumours and bioluminescence imaging of metastasis in vehicle- or celecoxib-treated mice (*n*=5). Scale bar, 200 μm. (**e**) VEGFC protein levels in MDA-MB-231 tumour cells in response to PGE2, measured by ELISA (*n*=5). (**f**) qRT–PCR analysis of *Cox2* (*Ptgs2*) expression in mouse primary macrophages (MØ) treated with isoproterenol (Iso)±propranolol (Prop) (*n*=3). (**g**) PGE2 production in mouse primary macrophages treated with vehicle or isoproterenol *in vitro*, measured by ELISA (*n*=3). (**h**) *Cox2* (*Ptgs2*) expression in CD11b^+^F4/80^+^ TAMs isolated from 66cl4 primary mammary tumours from control or stressed mice (*n*=5). (**i**) qRT–PCR analysis of tumour *Vegfc* gene expression and representative *in vivo* images of lung and LN metastasis in control or stressed mice with 66cl4 primary tumours (not shown, fourth mammary fat pad) 28 days post tumour cell injection treated with GW2580 or vehicle (*n*=5). All data represent mean±s.e. *P<0.05, **P<0.01 and ***P<0.001 by two-way analysis of variance (*post hoc* Tukey's adjustment) or Student's *t*-test.

While chronic stress increased tumour cell *VEGFC* expression in primary mammary tumours *in vivo* (Fig. 4a), direct treatment of cultured tumour cells with the β-adrenoceptor agonist isoproterenol was insufficient to drive *VEGFC* expression *in vitro* (Fig. 4f), suggesting that a mediating factor is required for the effect of stress on tumour cell *VEGFC* expression *in vivo*. As inflammation promotes VEGFC expression and drives lymphatic remodelling^15^, we investigated if inflammatory pathways are linked to stress regulation of lymphatic vasculature *in vivo.* We first looked at expression of the inflammatory marker cyclooxygenase-2 (*COX2*, or *PTGS2*). While tumour *COX2* levels were low in control mice, stress or isoproterenol elevated *COX2* mRNA transcript levels (Fig. 5a and Supplementary Fig. 5A), which were strongly associated with *VEGFC* mRNA levels (Fig. 5b and Supplementary Fig. 5B). This is consistent with findings in other solid cancers^49^,^50^,^51^,^52^, and extends observations that inflammation is linked with lymph vessel formation by identifying stress signalling as a potential driver of this relationship. To determine if inflammation is necessary for stress to modulate lymphatic architecture and lymphogenous spread, we treated control and stressed mice bearing MDA-MB-231 mammary tumours with the COX2 inhibitor celecoxib. Inhibition of COX2 activity blocked the effect of stress on tumour LYVE-1^+^ LVD (Fig. 5c,d) and prevented metastasis to lymph node and distant organs (Fig. 5c,d and Supplementary Fig. 5C), demonstrating a functional consequence of limiting lymphatic remodelling.

Prostaglandin E2 (PGE2) is a well-characterized inflammatory eicosanoid produced by COX2. To investigate if PGE2 may induce VEGFC in tumour cells, we treated MDA-MB-231 cells with PGE2. We found a concentration-dependent increase in tumour cell VEGFC expression and protein secretion (Fig. 5e and Supplementary Fig. 5D), suggesting the COX2-PGE2 inflammatory pathway as a plausible mediator of stress-induced lymphatic remodelling. Macrophages are a key source of inflammatory molecules in the tumour microenvironment^53^,^54^. They are also highly sensitive to stress signalling, which leads to their recruitment to primary mammary tumours^6^,^55^. To investigate if β-adrenergic signalling regulates macrophage PGE2 production, we treated primary macrophages with isoproterenol and assayed inflammatory mediators. Direct activation of β-adrenoceptor signalling elevated *COX2* expression and PGE2 production in primary bone marrow-derived macrophages (Fig. 5f,g) and human monocyte-derived macrophages (Supplementary Fig. 5E), while physiological activation of β-adrenoceptor signalling by chronic stress elevated *COX2* expression in CD11b^+^F4/80^+^ macrophages isolated from primary mammary tumours (Fig. 5h). These findings confirm that stress signalling can regulate the COX2-PGE2 pathway in tumour-associated macrophages (TAMs). To investigate if macrophages are necessary for the effects of stress on intratumoural lymphatic remodelling, we treated mice with a small molecule inhibitor of colony-stimulating factor 1 receptor GW2580, which we have previously shown blocks stress-induced macrophage recruitment to primary mammary tumours^6^. Blocking macrophage recruitment prevented stress-induced VEGFC expression in tumours and blocked lymphatic dissemination as shown by reduced lymph node metastasis (Fig. 5i). While not excluding other cellular sources of prostaglandins^56^, these data suggest that TAMs may be one source of the inflammatory signalling required for stress-enhanced *VEGFC* expression and lymphogenous dissemination.

### Clinical BB use is linked to reduced lymph node metastasis

**Figure 6:**
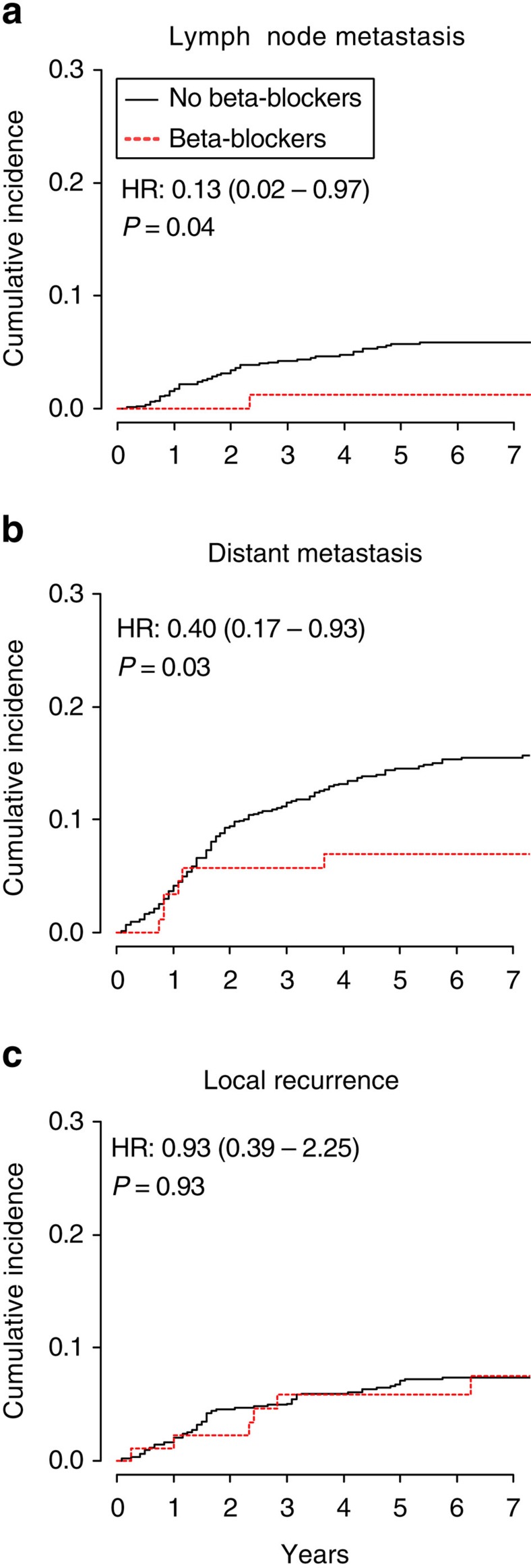
BB use is associated with reduced lymph node and distant metastasis. Cumulative incidence of (**a**) lymph node metastasis, (**b**) distant metastasis and (**c**) primary tumour recurrence by BB use in patients with triple-negative breast cancer (*n*=863, no BB; *n*=93, BB). HR, adjusted hazard ratio (95% confidence interval). *P* values calculated by Cox regression.

These findings suggest that targeting β-adrenergic stress-responsive signalling may provide a clinical strategy to limit lymphogenous tumour cell dissemination. Recent retrospective, epidemiological studies have linked BB use to improved cancer outcomes^57^,^58^,^59^,^60^, but it is unclear if lymphogenous dissemination was affected. We therefore investigated the incidence of lymph node metastasis in a cohort of 956 breast cancer patients during an average of 78 months after diagnosis (Table 1). Multivariate analysis revealed BB use was significantly associated with reduced risk of lymph node metastasis (Fig. 6a), after controlling for age, tumour stage, peritumoural vascular invasion, tumour treatment and other cardiac medications (hazard ratio, 0.13; 95% confidence interval, 0.02–0.97; *P*=0.04 by Cox regression analysis; Table 2). During the follow-up period, 47 lymph node metastases were identified in patients not taking BB (5.5% of patients) versus only 1 lymph node metastasis in patients taking BB (1.1%, *P*=0.07 by Gray's test; Table 2). Consistent with our earlier analysis of a subset of these patients^60^, BB use was also associated with decreased distant metastasis (Fig. 6b). There was no association between BB use and local recurrence (Fig. 6c), consistent with our *in vivo* studies that found β-adrenoceptor signalling did not affect primary tumour size or growth (Fig. 1c and Supplementary Fig. 1B).

## Discussion

**Figure 7:**
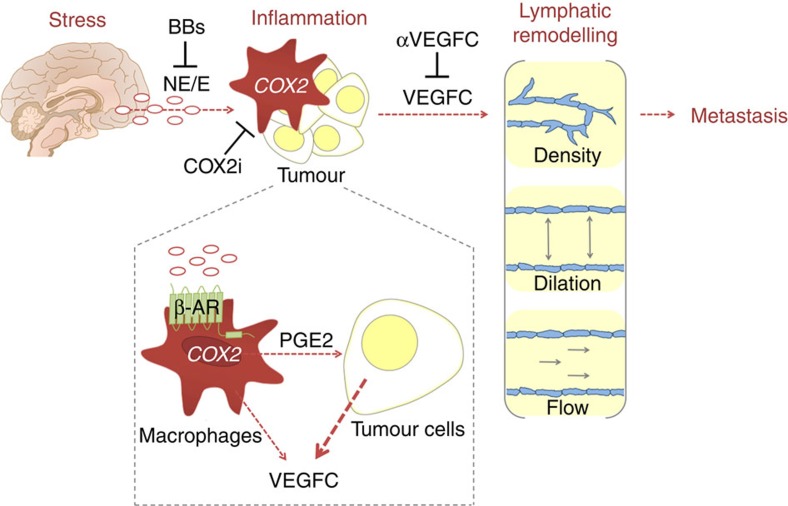
Stress-induced lymphatic remodelling. Stress remodels lymphatic vasculature through a tumour neural-inflammatory axis to promote lymphogenous tumour cell dissemination and metastasis. Tumour cell-derived VEGFC is necessary for stress-enhanced lymphatic remodelling but is not directly activated by β-adrenoceptor signalling. Tumour-associated macrophages respond to β-adrenoceptor signalling to produce inflammatory molecules such as PGE2, which may then signal to tumour cells to produce VEGFC required for lymphatic remodelling. These effects may be clinically blocked using BBs, anti-VEGFC therapeutics (αVEGFC) or COX2 inhibitors (COX2i). E, epinephrine; NE, norepinephrine; β-AR, β-adrenoceptor.

These findings identify chronic stress as a pathophysiological regulator of lymphatic remodelling in cancer (Fig. 7). We demonstrate that chronic stress induces stable changes in intratumoural and peritumoural lymphatic architecture, and increases lymphogenous tumour cell dissemination leading to increased lymph node metastasis. These studies define a critical role for neural-inflammatory signalling in the adverse effects of stress on tumour lymphatic remodelling. Additionally, the findings show that stress-induced vascular remodelling is dependent on β-adrenergic signalling to inflammatory cells, which increases tumour cell-derived VEGFC. Furthermore, we demonstrate the translational potential of these findings by showing that stress-induced SNS signalling may be blocked pharmacologically to prevent lymph vascular remodelling, to reduce lymph flow and to prevent tumour cell dissemination.

These findings expand our understanding of the role of lymphogenous dissemination in metastasis by identifying chronic stress as an external factor that drives lymphogenous spread. Lymphatic dissemination has been described as a passive process that presents the lowest barrier to tumour cell escape and survival^61^. Rather than being passive, we show that lymphatic dissemination may be directed by external (non-tumour) influences. Specifically, we show that chronic stress drives SNS signalling to form new lymphatic pathways of tumour cell escape. We show that SNS signalling increases flow through lymphatic vessels, which could potentially increase tumour cell dissemination. In addition, it has been suggested that increased lymph flow promotes immune tolerance of tumour antigens through a VEGFC-centric mechanism^62^, suggesting an alternate mechanism by which stress may promote metastatic escape by modulating the immune system. The importance of lymphatic dissemination in metastatic progression has been questioned due to the ability of distant metastasis to form seemingly in the absence of functional lymphatics^32^. Our studies using Patent Blue V drainage and intravital microscopy provide evidence for functional tumour-associated lymphatic vasculature and spontaneous tumour cell dissemination from orthotopic mammary tumours.

It will be important to define stromal sources of VEGFC (Fig. 4a). Macrophages are a known source of VEGFC^43^, and treatment with isoproterenol increases *Vegfc* expression in primary bone marrow-derived macrophages (Fig. 4f). However, we found no evidence that stress increased *VEGFC* expression by TAMs (Fig. 4g). This suggests that while macrophages may act through an autocrine mechanism to increase macrophage VEGFC synthesis, this may not be the main source of stromal VEGFC in tumours of stressed individuals. While it is also plausible that LECs and fibroblasts may be additional sources of prostaglandins^56^, we found that direct β-adrenergic signalling to LECs did not modulate PGE2 production *in vitro* (or *VEGFC* expression) (Fig. 4f and Supplementary Fig. 5E). In contrast, the data presented here suggest that PGE2 derived from TAMs may contribute to stress regulation of tumour cell-derived VEGFC. In addition, it will be important to define the direct effect of stress on lymphatic vasculature function, as isolated lymphatic vessels have been shown to respond to β-agonists^63^. Further studies using strategies such as cell-specific knockout will be required to assess the effect of β-adrenergic signalling on these various cell populations and determine their respective contributions to remodelling of lymphatic vasculature.

Our finding that BB treatment prevents lymphatic remodelling and tumour cell dissemination, provides new mechanistic insight to explain recent clinical observations that BB use is associated with reduced metastasis and improved survival^57^,^58^,^59^,^60^. β-adrenergic regulation of lymphogenous tumour cell dissemination—with no effect on primary tumour growth—may explain why clinical BB use was not associated with primary tumour recurrence. Our finding that SNS signalling may be acutely leveraged to limit lymph flow suggests that the perioperative period may be an additional opportunity for interventions that target stress signalling. Selective choice of anaesthetic agents that block SNS signalling and lymph flow may help to limit dissemination of tumour cells that become displaced from the tumour during surgical resection. While the capacity of anaesthetic sympathectomy to limit tumour cell dissemination or perioperative inflammation is still unclear, the use of SNS-blocking neuraxial anaesthesia has been associated with improved cancer outcomes^64^.

These findings suggest that it may be important to identify stressed individuals who may be particularly susceptible to lymphogenous dissemination. One approach may be through transcriptional profiling using a stress signature^55^. Alternatively, as cancer is often a highly stressful experience, it is plausible that SNS intervention may be generally useful to improve cancer outcome. In support of that contention, we found here that clinical BB use was linked to a significant reduction in lymph node metastasis (and reduced distant metastasis) in a cancer cohort without prior evaluation of stress levels.

Stress regulation of lymphatic vasculature may have evolved to promote survival during times of threat. Co-ordinated regulation of the fight-or-flight stress response with increased lymphatic function may have provided an evolutionary advantage by enhancing immune surveillance and activating a rapid immune response to physical threat. However, the findings presented here demonstrate that SNS-regulated lymphatic function can have adverse effects in the context of chronic diseases such as cancer. Importantly, these findings identify multiple points of clinical intervention to limit these adverse effects of stress.

## Methods

### Cells

A highly metastatic variant of the MDA-MB-231 triple-negative breast adenocarcinoma cell line (MDA-MB-231HM, a kind gift from Dr Zhou Ou, Fudan University Shanghai Cancer Center, China) was transduced with a lentiviral vector containing codon-optimized firefly luciferase-eGFP or -mCherry under the control of the ubiquitin-C promoter as previously described^65^, and was cultured in DMEM medium (Invitrogen) containing 10% fetal bovine serum (FBS) and 200 mM glutamine. The identity of this cell line was confirmed by karyotyping (Cellbank, Australia). Luminal B 66cl4 mammary adenocarcinoma cell line (a kind gift from Prof. Robin Anderson, Peter MacCallum Cancer Centre, Australia) was transduced with FUhlucW construct as previously described^6^, and cultured in α-MEM medium (Invitrogen) containing 10% FBS and 200 mM glutamine. Primary mouse macrophages were derived from bone marrow of BALB/c *nu/nu* mice. Bone marrow was isolated from mouse femurs and then cultured in RPMI medium (Invitrogen) containing 10% FBS, 200 nM glutamine and 100 ng ml^−1^ of CSF-1 (Sigma) for 7 days for macrophage maturation. Human monocyte cell line U937 (American Type Culture Collection)^66^ was cultured in RPMI medium (Invitrogen) containing 10% FBS and 200 nM glutamine. To induce differentiation into macrophages, U937 cells were treated with phorbol 12-myristate 13-acetate (200 nM, Sigma) for 24 h. Human lymphatic endothelial cells (human dermal microvascular endothelial cells; HMVEC-D, catalogue #: CC-2812, Lonza) were cultured in EGM-2MV BulletKit supplemented with 5% FBS (Lonza). All cells were maintained at 37 °C and 5% CO_2_. *VEGFC* was silenced in MDA-MB-231 cells by stable expression of short hairpin RNA against *VEGFC* (TRCN0000058503, Mission Viral Particles, Sigma). Cells were transduced with viral particles (multiplicity of infection 2) for 5 h and selected with puromycin (8 μg ml^−1^; Invitrogen). Single-cell clones were established from GFP^+^ sorted cells, and knockdown of mRNA and protein levels were confirmed by qRT–PCR and ELISA (R&D Systems), respectively (Supplementary Fig. 4D). All cells were confirmed negative for mycoplasma contamination before use using the MycoAlert Mycoplasma Detection Kit (Lonza).

### Breast cancer models

For orthotopic tumour studies, 2 × 10^5^ MDA-MB-231 cells or 1 × 10^5^ 66cl4 cells in 20 μl PBS (Invitrogen) were injected into the fourth mammary fat pad of anaesthetized (3% isoflurane) 8-week-old, female BALB/c *nu/nu* (University of Adelaide, Australia) or BALB/cJAsmu (Monash Animal Research Platform, Australia) mice, respectively. Mice were housed under PC2 barrier conditions on 12 h dark/light cycle. Primary tumours were measured by caliper, and volume was calculated by the formula: (length × width^2^)/2. Metastasis was tracked with bioluminescence using an IVIS Lumina II (Perkin Elmer) imaging system by measuring luciferase activity (60-s exposure) after a tail-vein injection of 150 mg kg^−1^ D-luciferin (CHOICE Analytical). After 22 (MDA-MB-231) or 29 (66cl4) days of tumour progression, mice were killed for evaluation. Tissue-specific metastasis in lung, liver and lymph nodes were imaged *ex vivo* and frozen in liquid nitrogen for immunohistochemistry and gene expression studies. For the spontaneous metastasis model, 7-week-old female MMTV-PyMT-C57Bl/6 mice^34^ were used and metastasis evaluated by histology. CD11b^+^F4/80^+^ TAMs were isolated from 66cl4 mammary tumours by fluorescence-activated cell sorting. Primary tumours were dissociated using collagenase type IV (Gibco) and stained with fluorescent-conjugated antibodies against CD11b (BD Biosciences, clone M1/70, 1:200) and F4/80 (eBioscience, Clone BM8, 1:200). Sample sizes were determined using conservative sample size calculations under a mixed-effect longitudinal model with a power of >0.90 to detect a 5% change compared to control at the 0.05 significance level. All procedures were carried out under protocols approved by the Monash Institute of Pharmaceutical Sciences Animal Ethics Committee.

### Chronic restraint stress

Female BALB/c *nu/nu*, BALB/cJAsmu or MMTV-PyMT-C57Bl/6 mice at 8 weeks of age were randomly assigned to home cage control conditions or chronic restraint stress (*n*=5 per experimental group). Mice were restrained in a confined space that prevented them from moving freely but did not press on them. These conditions increase tissue levels of the stress neurotransmitter norepinephrine^3^. Mice were restrained for 2 h per day for 21 days commencing 7 days before tumour cell inoculation or from 8 weeks of age for the MMTV-PyMT model.

### Pharmacologic studies

For β-agonist studies, 8-week-old female BALB/c *nu/nu* mice received isoproterenol (Sigma; 5 mg kg^−1^ day^−1^) by daily subcutaneous injections for a total of 21 days. For BB studies, mice received propranolol (Sigma; 5 mg kg^−1^ day^−1^) by mini-osmotic pump (Alzet, model 1004). For macrophage inhibition studies, mice received GW2580 (R.I. Chemical; 160 mg kg^−1^) as previously described^6^. For COX2 inhibitor studies, mice received celecoxib (Celebrex; Clifford Hallam; 25 mg kg^−1^ day^−1^) in drinking water. Mice were treated with inhibitors for the duration of the study.

### Lymphangiography

A volume of 20 μl of 0.25% (w/v) Patent Blue V dye (Sigma) was injected intratumourally in anaesthetized 8-week-old female BALB/c *nu/nu* mice for uptake into collecting lymphatic vessels as previously described^28^. A skin flap preparation was conducted by making an incision along the central line separating the peritoneum from the skin containing lymphatic collectors. Draining-collecting lymphatic vessels were imaged using a dissecting microscope fitted to an AxioCam ERc 5s camera (Zeiss). Average vessel diameter was quantified across ⩾5 intervals using the Fiji distribution of ImageJ software (NIH) by an investigator blinded to treatment conditions.

### Immunohistochemistry

Following antigen retrieval with citrate buffer (0.01 M, pH 6), 5 μm cryosections of primary tumours or lymph nodes were fixed with 4% paraformaldehyde (Sigma) and then incubated with one or more primary antibodies for 16 h at 4 °C. Anti-LYVE-1 (Fitzgerald, catalogue #: 70R-LR003, 1:500) antibody was used to localize lymphatic vasculature followed by incubation with corresponding Alexa-Fluor-conjugated secondary antibody (Invitrogen, catalogue #: A31573, 1:500) and Hoechst 33342 nuclear staining (Sigma, catalogue #: B2261, 1:1000). Immunostaining was visualized at × 40 magnification using an Eclipse 90i microscope (Nikon). LYVE-1^+^ LVD was evaluated in ⩾5 digital images per sample captured at random intervals around primary tumour margins^32^ by an investigator blinded to treatment conditions. Area of vessel density (percentage of total tissue area) was quantified using ImageJ software.

### *In vivo* lymph flow

Inert Fluoresbrite YG carboxylate nanospheres (50 nm; Polysciences) at 0.025% in 10 μl PBS were injected into the left inguinal lymph node of anaesthetized 8-week-old female BALB/c *nu/nu* mice. Visualization of nanospheres was carried out using a Leica SP8 multiphoton as described above. Emission was collected at 455/25 (second harmonic generation), 525/25 (nanospheres) and 585/20 nm (mCherry tumour cells). The baseline rate of flow of nanospheres through the efferent lymphatic vessel was quantified after 3 min had elapsed to allow flow to stabilize. To investigate stress signalling in anaesthetized mice, stress neurotransmitter norepinephrine (Sigma; 1 mg kg^−1^) was injected intravenously and impact on lymph flow investigated. To avoid confounding of anatomical variation, lymph flow readings were taken in each mouse before and after norepinephrine administration. Quantification of lymphatic flow rate was carried out by manually tracking individual particles using the Fiji distribution of ImageJ over ⩾10 time points. The average velocity of each particle was then calculated in μm s^−1^.

### Nuclear lymphoscintigraphy

This proof-of-concept study involved comparing lower limb lymphatic drainage under resting conditions with that following sympathetic blockade in a consenting patient receiving brachytherapy for cervical carcinoma (Project Number 09/15, Human Research Ethics Committee Peter MacCallum Cancer Centre; ACTRN12612001162808). Written, informed consent was obtained from the study subject. Regional sympathetic blockade was induced by intrathecal ropivicaine administered at the L3/4 interspace. Confirmation of spinal anaesthesia was made before commencement of the lymphoscintigram using ice testing to ensure a dermatome block higher than T_9_ (ref. 67). Unlike most patients receiving spinal anaesthesia, patients receiving brachytherapy have no surgery-induced lower limb wound oedema that would otherwise confound the study's results; patients with pre-existing alterations in lymphatic flow or sympathetic tone, chronic regional pain syndrome, lymphoedema, surgery or trauma to the lower limbs, obesity (body mass index >30 kg m^−2^), diabetes mellitus, patients receiving adrenergic receptor antagonists or anti-inflammatory agents, and clinical pelvic or lower limb lymphadenopathy were excluded. To minimize the confounding of anatomical variation, lymph flow was assessed before and after the implementation of sympathetic blockade in the same individual. In alliance with common anaesthesia practice, intravenous fluid resuscitation was provided intraoperatively to the patient to achieve restoration of blood pressure within 20% of starting blood pressure. ^99m^Tc-ASC (0.25 ml), a colloid that migrates through the lymphatic vessels and is retained in the sentinel lymph node for 24 h until cleared, was injected sub-dermally in the first web-space of both feet, and tracked from feet to inguinal lymph nodes using dynamic image acquisition over 60 min (two head gamma camera, Philips Skylight).

### Secreted protein quantification

Cells were serum-starved overnight before treatment with isoproterenol or PGE2 (Sigma). After 24 or 72 h conditioned medium was centrifuged at 2,000 r.p.m. for 5 min. Secreted levels of VEGFC or PGE2 were quantified by ELISA according to the manufacturer's instructions (R&D Systems).

### Messenger RNA isolation and qRT–PCR

qRT–PCR using TaqMan probes was used to quantify *VEGF* (*VEGFA*; Hs00900055_m1), *VEGFC* (Hs01099203_m1), *FIGF* (*VEGFD*; forward, 5′- GTATGGACTCTCGCTCAGCAT -3′; reverse, 5′- AGGCTCTCTTCATTGCAACAG -3′), *Ptgs2* (*Cox2*; Mm00478374_m1), *PTGS2* (*COX2*; Hs00153133_m1), *Flt4* (*Vegfr3*; Mm01292604_m1), *FLT4* (*VEGFR3*; Hs01047677_m1), *Rpl30* (Mm01611464_g1) and *RPL30* (Hs00265497_m1). Total RNA was isolated from 2 mm^3^ of mammary primary tumour tissue or 10^6^ cultured cells using RNeasy Mini Kit. A unit of 100 ng total RNA was assayed by qRT–PCR reactions using standard PCR conditions through 60 PCR amplification cycles and normalized to human or mouse *RPL30* housekeeping gene.

### Clinical beta-blockade

A detailed description of a cohort of 800 triple-negative breast cancer patients diagnosed and operated at the European Institute of Oncology in Milan between 1997 and 2008 has been previously described^60^. For this present study we added 2 years of observation (156 patients operated in the same Institute between 2009 and 2010) and updated the entire cohort follow-up. Use of the data, retrieved from the European Institute of Oncology Breast Cancer Institutional Database, was approved by the European Institute of Oncology Review Board. At the time of admission to the hospital, all patients gave their consent for the use of their data for research purposes. Patients with history of any previous invasive cancer or with metastatic disease at diagnosis (stage IV) were previously excluded. Patients were divided into two groups: the BB users group, which included patients who were using any BB at the time of cancer diagnosis; and the BB non-users group, which included all other patients. Association between the occurrence of first metastatic events and BB use was evaluated in a competing-risk survival analysis framework. Definition of events: local events are recurrences in the ipsilateral breast. Lymph node events are events that involve the axillary lymph nodes or the regional lymph nodes (that is, internal mammary chain lymph nodes or supraclavicular lymph nodes). Distant events are metastases in distant organs.

### Statistical analysis

All bar graphs show sample means±s.e. Single comparisons were performed using unpaired two-sided Student's *t*-test without equal variance. Multiple comparisons were performed using one-way analysis of variance or two-way analysis of variance with Dunnett's or Tukey's correction for multiple comparisons, respectively, and were confirmed using non-parametric Mann–Whitney *U*-tests. Comparisons between Kaplan–Meier curves were performed using log-rank test. Statistical significance was assumed where *P*<0.05. A generalized linear model approach based on beta regression was used to model the measured density of LYVE-1 immunostaining in primary tumour sections^68^. Since the response measurements occur in the interval between 0 and 1, we model the response measurements *Y*_i_ by a beta distribution, beta(*μ*_i_, Φ_i_), where *μ*_i_ is the mean response and *μ*_i_(1−*μ*_i_)/Φ_i_ is the response variance. In our model both the mean response and the dispersion parameter depend on a linear combination of the main treatment effects, with the dispersion parameter capturing mouse heterogeneity. Model parameters were fitted using maximum likelihood estimation. Results are reported in Supplementary Table 1.

Association between categorical variables and BB use was evaluated by *χ*^2^-test, Fisher exact test or *χ*^2^-test for trend, as appropriate. Differences in median age and Ki-67 were tested using the non-parametric median two-sample test. Cumulative incidences of breast cancer outcomes were compared across different patient subgroups using the Gray test^69^. Multivariable Cox proportional hazard models were applied to adjust the effect of BB for age, tumour stage, peritumoural vascular invasion, tumour treatment, other antihypertensives, antithrombotics and statins^60^. Adjusted hazard ratios with 95% confidence intervals were reported. All analyses were carried out with SAS software (SAS Institute, Cary, NC) and R software, version 2.12.2 (ref. 70). All *in vitro* and *in vivo* experiments were repeated 2–4 times.

## Additional information

**How to cite this article:** Le, C. P. *et al*. Chronic stress in mice remodels lymph vasculature to promote tumour cell dissemination. *Nat. Commun.* 7:10634 doi: 10.1038/ncomms10634 (2016).

## Supplementary Material

Supplementary Information Supplementary Figures 1-5 and Supplementary Figures 1-2.

Supplementary Movie 1 3D reconstruction of a multi-photon image showing collecting lymphatic vessel containing fluorescent carboxylate nanospheres (green) and mCherry-tagged MDA-MB-231 tumor cells (red) running adjacent to a blood vessel.

Supplementary Movie 2 Flow of fluorescent carboxylate nanospheres (green) in a lymph vessel visualized with multi-photon imaging before (control, top) and after norepinephrine treatment (stress, bottom). The video is encoded in real time. Scale bar: 20 μm.

## Figures and Tables

**Table 1:** Characteristics at baseline by BB intake.

**Variable**	**Category**	**No BB no. (%)**	**BB no. (%)**	***P*** **value**
Total		863 (90.3)	93 (9.7)	
Age	Median (range)	60 (30–92)	63 (48–80)	<0.01
	<65	593 (68.7)	50 (53.8)	<0.01
	⩾65	270 (31.3)	43 (46.2)	
BMI	<25	459 (59.8)	48 (53.9)	0.10
	25–30	238 (31.0)	27 (30.3)	
	>30	70 (9.1)	14 (15.7)	
T*	1	387 (46.0)	51 (54.8)	0.23
	2	340 (40.4)	31 (33.3)	
	3	42 (5.0)	4 (4.3)	
	4	72 (8.6)	7 (7.5)	
N*	0	449 (54.4)	51 (56.0)	0.89
	1	253 (30.7)	26 (28.6)	
	2/3	123 (14.9)	14 (15.4)	
Ki-67	Median (range)	42 (1–95)	38 (4–90)	0.12
	≤40	408 (49.2)	53 (57.6)	0.12
	>40	422 (50.8)	39 (42.4)	
Peritumoural vascular invasion	Absent	650 (75.8)	73 (79.3)	0.39
	Focal	110 (12.8)	11 (12.0)	
	Extensive	98 (11.4)	8 (8.7)	
Neoadjuvant chemotherapy	No	716 (83.0)	80 (86.0)	0.45
	Yes	147 (17.0)	13 (14.0)	
Type of surgery	MAST	206 (23.9)	20 (21.5)	0.61
	QUAD	657 (76.1)	73 (78.5)	
Radiotherapy	No	138 (16.0)	12 (12.9)	0.44
	Yes	725 (84.0)	81 (87.1)	
ACEI	No use	763 (88.4)	68 (73.1)	<0.01
	Use	100 (11.6)	25 (26.9)	
ARB	No use	817 (94.7)	80 (86.0)	<0.01
	Use	46 (5.3)	13 (14.0)	
CCB	No use	808 (93.6)	79 (84.9)	<0.01
	Use	55 (6.4)	14 (15.1)	
Diuretic	No use	785 (91.0)	60 (64.5)	<0.01
	Use	78 (9.0)	33 (35.5)	
Antithrombotics	No use	837 (97.0)	83 (89.2)	<0.01
	Use	26 (3.0)	10 (10.8)	
Statins	No use	812 (94.1)	80 (86.0)	<0.01
	Use	51 (5.9)	13 (14.0)	

ACEI, angiotensin-converting enzyme inhibitor; ARB, angiotensin receptor blocker; BMI, body mass index; CCB, calcium channel blocker; MAST, mastectomy; QUAD, quadrantectomy.

^*^Clinical stage was used for patients undergoing neoadjuvant chemotherapy. Numbers might not sum up to totals due to missing values. *P* values were obtained using *χ*2-test or Fisher's exact test, as appropriate.

**Table 2:** Events and follow-up by BB intake.

**Event**	**No BB no. (%)**	**BB no. (%)**	***P*** value*	**HR (95% CI)**†	***P*** **value**†
*At risk*	863	93			
					
*First events*					
Lymph node metastasis	47 (5.5)	1 (1.1)	0.07	0.13 (0.02–0.97)	0.04
Distant metastasis	131 (15.2)	6 (6.5)	0.04	0.40 (0.17–0.93)	0.03
Local recurrence	68 (7.9)	6 (6.5)	0.74	0.93 (0.39–2.25)	0.93
Local or regional or distant	246 (28.5)	13 (14.0)	<0.01	0.48 (0.25–0.79)	<0.01
					
*Overall mortality*					
Death from breast cancer	161 (18.9)	8 (8.6)	0.03	0.48 (0.23–0.99)	0.05
Death from other or missing causes	48 (5.6)	10 (10.8)	0.02	1.75 (0.84–3.62)	0.13
					
*Follow-up*					
Months, median (Q1–Q3)	78 (46–109)	77 (47–105)	0.91		

CI, confidence interval; HR, hazard ratio; Q1, lower quartile; Q3, upper quartile.

^*^Univariate analysis: association between number of events and BB use was tested by the Gray test, while association between follow-up and BB use was tested by the median two-sample test.

^†^HRs and *P* values for testing BB versus non-BB were obtained using a Cox regression multivariable model that included age, tumour size, lymph node status, peritumoural vascular invasion, type of surgery, radiotherapy, other cardiac medications and statins.
