# Supplementary material for: Chronic stress in mice remodels lymph vasculature to promote tumour cell dissemination
Source: Nat Commun. 2016 Mar 1;7:10634. doi: 10.1038/ncomms10634 (PMC4773495; doi:10.1038/ncomms10634)
Supplement: Supplementary Information — Supplementary Figures 1-5 and Supplementary Figures 1-2. [file ncomms10634-s1.pdf]

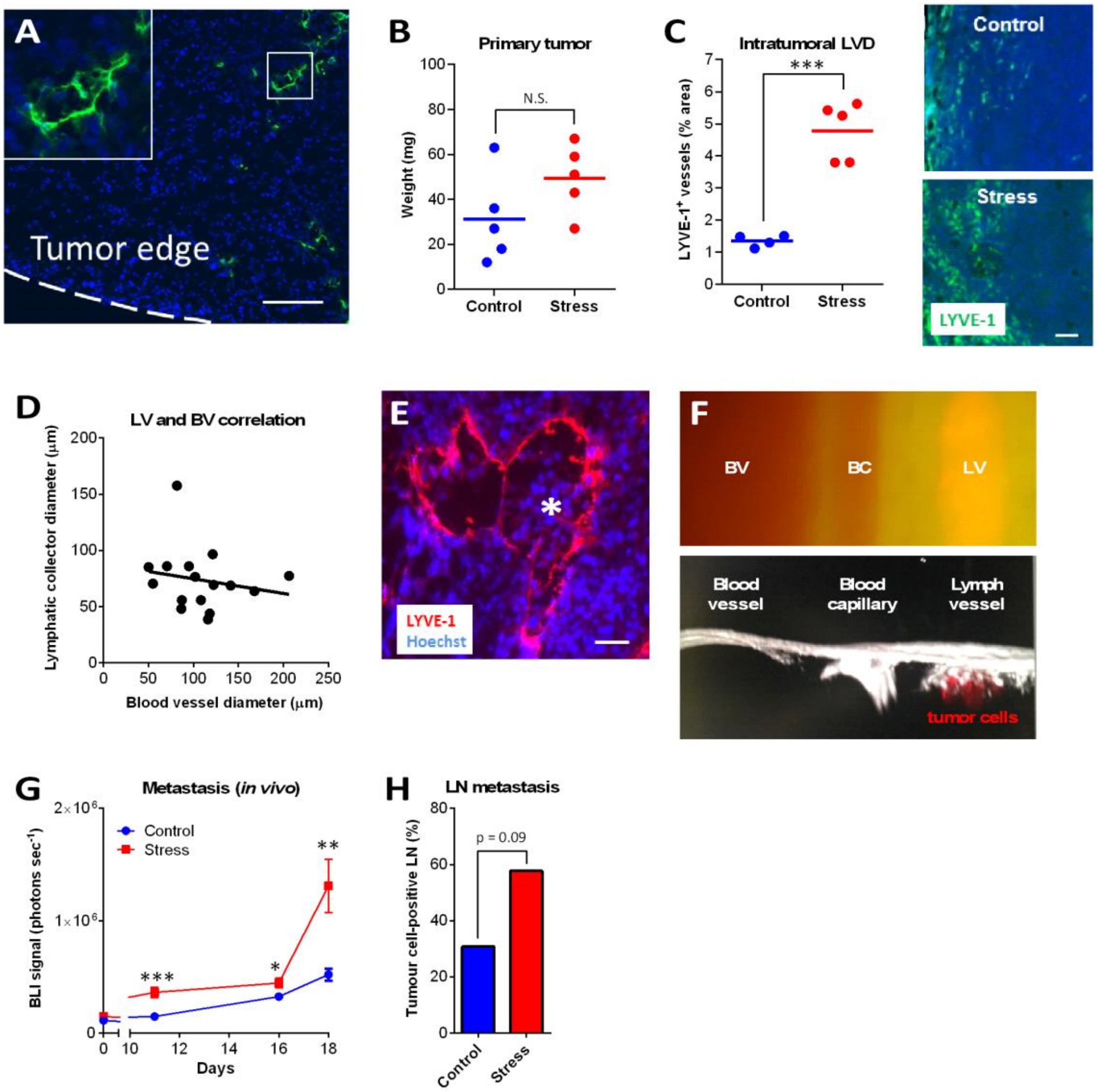

Supplementary Figure 1. Chronic stress remodels tumor lymphatic architecture (A) Representative image of LYVE-1$^+$ vessel staining (inset) in topographical relation to the tumor edge (dashed line). Scale bar: 100 µm (inset; 20 µm). **(B)** Mass of MDA-MB-231 primary mammary tumors of control versus stressed mice (n = 5). N.S. = not significant. **(C)** Quantification of LVD (LYVE-1$^+$, green) and representative immunostaining in 66cl4 primary tumors of control or stressed mice. Blue: nuclear staining. Data represent mean of >8 fields of view (n = 5). **(D)** Correlation of lymphatic vessel diameter against blood vessel diameter. **(E)** Cell embolus (asterisk) in LYVE-1$^+$ (red) lymphatic vessel. Blue: nuclear staining. Scale bar: 200 µm. **(F)** Top: Representative fluorescent image of collecting lymphatic vessel (LV) containing mCherry-tagged MDA-MB-231 tumor cells (orange) and adjacent autofluorescent blood capillary (BC) and blood vessel (BV). Bottom: Cross-sectional view of a maximum intensity projection of the multiphoton image. Tumor cells: red. **(G)** Quantification of bioluminescence imaging in control versus stressed mice with MDA-MB-231 tumors up to 18 days after tumor cell (n = 5 at each timepoint). **(H)** Frequency of tumor-cell positive lymph nodes (LN) in control versus stressed mice (n = 13). All data represent mean ± SE. * $p < 0.05$, **$p < 0.01$, ***$p < 0.001$ by Student's t-test.

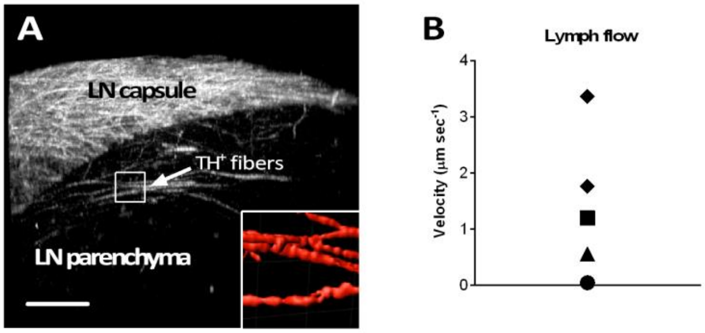

Supplementary Figure 2. Lymph node innervation and measurement of lymphatic flow (A) Multi-photon image of a lymph node innervated by TH^+ sympathetic nerve fibers from a C57Bl/6-TH-mCherry mouse. The arrow indicates the location of TH^+ fibers within the lymph node parenchyma and dense innveration of the lymph node (LN) capsule. Scale bar: 100 µm. The inset represents a space filling model showing varicosities that release NE. Scale: 20 µm. **(B)** Quantification of baseline lymph velocity in BALB/c *nu/nu* mice (n = 5).

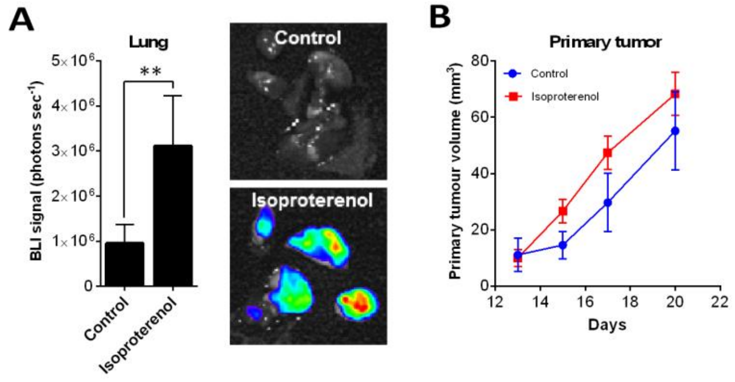

Supplementary Figure 3. Beta-adrenergic signaling increases metastasis (A) Representative images of lung metastasis and quantification of lung bioluminescence in control or isoproterenol treated mice (n = 5). **p < 0.01 by Student's t-test. **(B)** Primary mammary tumor volume of control or isoproterenol treated mice over time. Data represent mean ± SE (n = 5 at each timepoint).

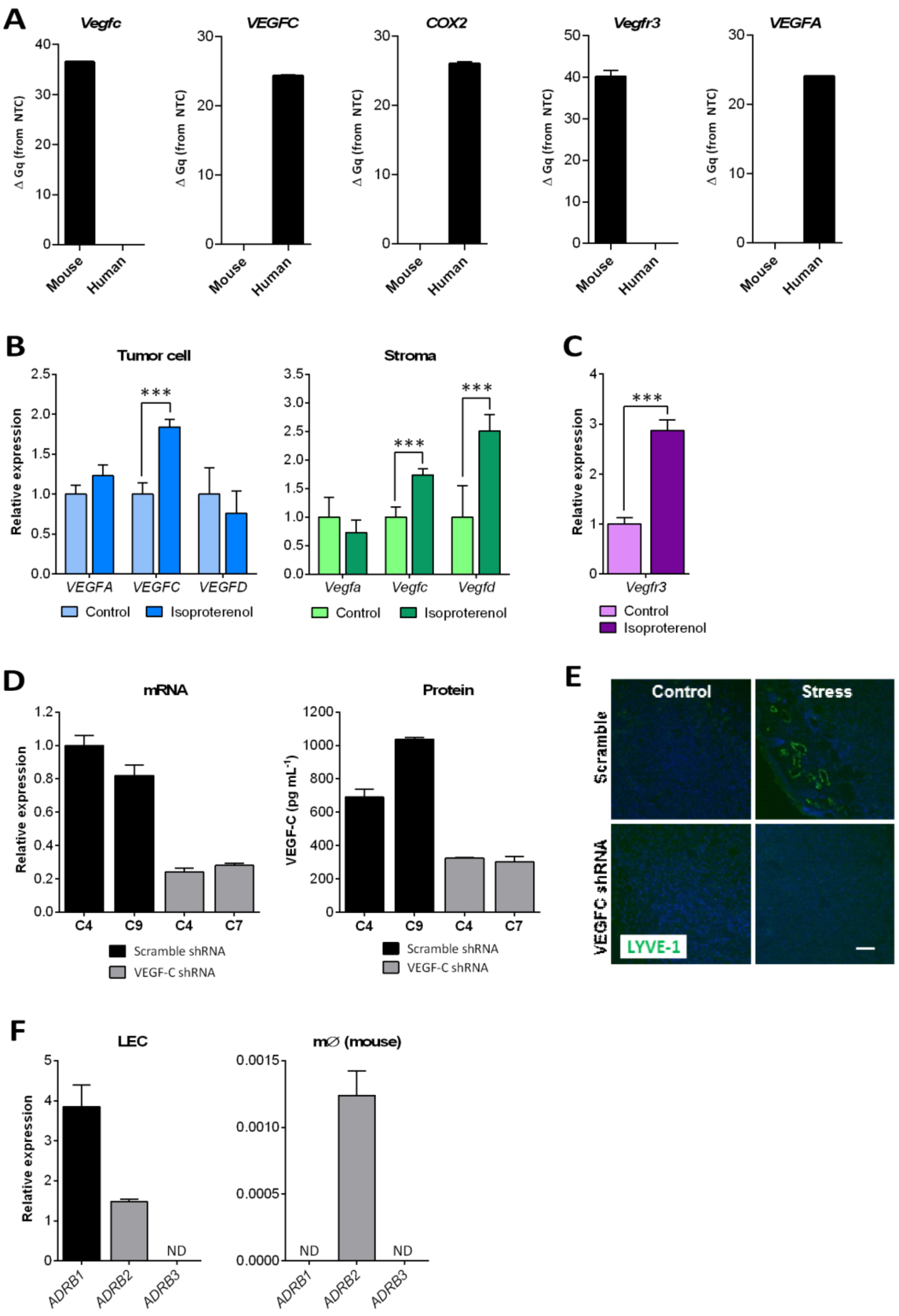

Supplementary Figure 4. VEGFC mediates stress-induced lymphatic remodeling (A) Specificity of PCR probes against 66cl4 (mouse) and MDA-MB-231 (human) cells. **(B)** qRT-PCR analysis of human-specific (tumor cell) and mouse-specific (stromal) lymphangiogenesis-linked gene expression in MDA-MB-231 primary tumors (n = 5). **(C)** qRT-PCR analysis of mouse-specific *Vegfr3* (*Flt4*) gene expression in MDA-MB-231 primary tumors (n = 5). **(D)** Quantitative analysis of *VEGFC* gene expression (left) and protein levels (right) in MDA-MB-231 clones treated with scramble (SCR) or *VEGFC* (KD) shRNA (n = 3). **(E)** Representative LYVE-1 (green) and nuclear (blue) staining of MDA-MB-231 tumors expressing scramble or *VEGFC* shRNA. Scale bar: 100 µm. **(F)** qRT-PCR analysis of *ADRB-1, -2 and -3* gene expression in human lymphatic endothelial cells (LECs) and primary mouse bone-marrow derived macrophages. ND = not detected. (n = 3). All data represent the mean ± SE. *p < 0.05 and ***p < 0.001 by two-way ANOVA on log transformed data (*post hoc* Tukey's adjustment) or Student's t-test.

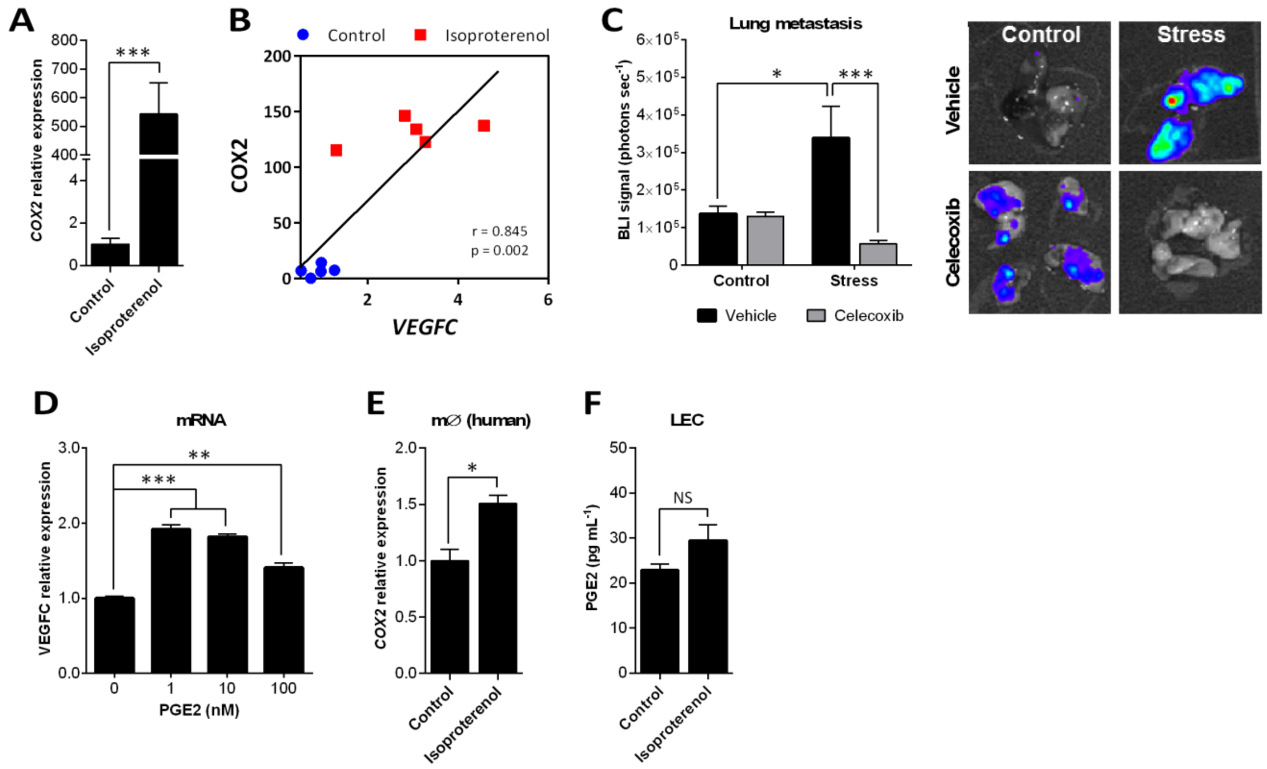

Supplementary Figure 5. Stress remodels tumor lymphatics via inflammation (A) qRT-PCR analysis of *COX2* gene expression in CD11b^{+}F4/80^{+} tumor-associated macrophages (TAMs) isolated from 66cl4 tumors from mice treated with vehicle or isoproterenol (n = 3). **(B)** Quantitative analysis of relationship between *COX2* and *VEGFC* expression in MDA-MB-231 tumors of control or isoproterenol-treated mice. Pearson coefficient 0.845 (p = 0.002, n = 5) **(C)** *Ex vivo* quantification and representative bioluminescence images of lung metastasis at day 22 of MDA-MB-231 tumor progression from celecoxib treated control or stressed mice (n = 5). **(D)** qRT-PCR analysis of *VEGFC* gene expression in MDA-MB-231 cells treated with increasing concentrations of PGE2. (n = 3). **(E)** qRT-PCR analysis of *COX2* gene expression in U937 monocyte-derived macrophages in response to vehicle or isoproterenol (n = 3). **(F)** PGE2 production in lymphatic endothelial cells (LECs) treated with vehicle or isoproterenol *in vitro* measured by ELISA. All data represent mean ± SE. $^{*}p < 0.05$, $^{**}p < 0.01$ and $^{***}p < 0.001$ by Two-way ANOVA test (*post hoc* Tukey's adjustment) or Student's t-test.

Supplementary Tables

	**Mean coefficients** (μ_i)	**Dispersion coefficients** (Φ_i)
Intercept	-4.75(0.30)*	3.48(0.35)*
Stress	0.86(0.42)*	-0.97(0.48)*
Control + Propranolol	0.13(0.44)	-0.20(0.50)
Stress + Propranolol	0.66(0.43)	0.71(0.49)

Supplementary Table 1. Estimated coefficients for the beta regression model of the effect of stress and BB (propranolol) on LYVE-1 immunostaining. Beta (μ_i, Φ_i) where μ_i is the mean LVD, and $\mu_i(1-\mu_i)/\Phi_i$ is the variance in LVD across individual primary tumor sections. Both μ_i and Φ_i are modeled as linear combinations of the treatment effects. Estimates are obtained by maximum likelihood estimation. Standard errors are reported in parenthesis and with (*) denoting that the coefficient is significantly different from 0 at the 5% significance level using a Z-test.

	**LN positive**	**LN negative**	**TOTAL**
**Control**	8	18	26
**Stress**	15	11	26
**TOTAL**	23	29	52

Supplementary Table 2. Tumor cell positive lymph node status in control versus stressed mice. Significance of association between stress and lymph node (LN) positivity for tumor cells. p = 0.093 determined using Fisher's exact test.
